# Cost-effectiveness of the national dog rabies prevention and control program in Mexico, 1990–2015

**DOI:** 10.1371/journal.pntd.0009130

**Published:** 2021-03-04

**Authors:** Jesús Felipe González-Roldán, Eduardo A. Undurraga, Martin I. Meltzer, Charisma Atkins, Fernando Vargas-Pino, Verónica Gutiérrez-Cedillo, José Ramón Hernández-Pérez

**Affiliations:** 1 Centro Nacional de Programas Preventivos y Control de Enfermedades (CENAPRECE), Secretaría de Salud México, Ciudad de México, México; 2 Escuela de Gobierno, Pontificia Universidad Católica de Chile, Santiago, Región Metropolitana, Chile; 3 Millennium Initiative for Collaborative Research in Bacterial Resistance (MICROB-R), Santiago, Región Metropolitana, Chile; 4 National Center for Emerging and Zoonotic Infectious Diseases, Centers for Disease Control and Prevention, Atlanta, Georgia, United States of America; 5 Subdirección de Rabia y otras Zoonosis del CENAPRECE, Ciudad de México, México; 6 Coordinador Estatal de Zoonosis, Secretaría de Salud Tlaxcala, México; Khon Kaen University, THAILAND

## Abstract

**Background:**

Rabies is a viral zoonosis that imposes a substantial disease and economic burden in many developing countries. Dogs are the primary source of rabies transmission; eliminating dog rabies reduces the risk of exposure in humans significantly. Through mass annual dog rabies vaccination campaigns, the national program of rabies control in Mexico progressively reduced rabies cases in dogs and humans since 1990. In 2019, the World Health Organization validated Mexico for eliminating rabies as a public health problem. Using a governmental perspective, we retrospectively assessed the economic costs, effectiveness, and cost-effectiveness of the national program of rabies control in Mexico, 1990–2015.

**Methodology:**

Combining various data sources, including administrative records, national statistics, and scientific literature, we retrospectively compared the current scenario of annual dog vaccination campaigns and post-exposure prophylaxis (PEP) with a counterfactual scenario without an annual dog vaccination campaign but including PEP. The counterfactual scenario was estimated using a mathematical model of dog rabies transmission (*RabiesEcon*). We performed a thorough sensitivity analysis of the main results.

**Principal findings:**

Results suggest that in 1990 through 2015, the national dog rabies vaccination program in Mexico prevented about 13,000 human rabies deaths, at an incremental cost (MXN 2015) of $4,700 million (USD 300 million). We estimated an average cost of $360,000 (USD 23,000) per human rabies death averted, $6,500 (USD 410) per additional year-of-life, and $3,000 (USD 190) per dog rabies death averted. Results were robust to several counterfactual scenarios, including high and low rabies transmission scenarios and various assumptions about potential costs without mass dog rabies vaccination campaigns.

**Conclusions:**

Annual dog rabies vaccination campaigns have eliminated the transmission of dog-to-dog rabies and dog-mediated human rabies deaths in Mexico. According to World Health Organization standards, our results show that the national program of rabies control in Mexico has been highly cost-effective.

## Author summary

Rabies is a virus that affects wild and domestic animals that can affect humans. Rabies infection is almost certainly fatal once clinical symptoms appear but can be prevented with timely post-exposure prophylaxis administration. Dogs are the primary source of rabies transmission; eliminating dog rabies reduces the risk of exposure in humans significantly. In Mexico, the national program of dog rabies vaccination has accomplished a progressive reduction of rabies episodes since 1990. The impact of these campaigns has not been systematically assessed, and that evaluation is critical to inform public health decisions. Using a mathematical model of rabies transmission, we estimated the costs and effectiveness of the dog rabies vaccination program in Mexico, compared to a scenario with no vaccination. Our results suggest that from 1990 through 2015, the national dog rabies vaccination program in Mexico prevented about 13,000 human rabies deaths, at an additional cost (MXN 2015) of $4,700 million (USD 300 million). We estimated an average cost of $360,000 (USD 23,000) per human rabies death averted, $6,500 (USD 410) per additional year-of-life, and $3,000 (USD 190) per dog rabies death averted. According to World Health Organization standards, the annual dog vaccination campaign in Mexico is highly cost-effective.

## Introduction

Rabies is a viral zoonosis that imposes a high disease burden in many developing countries [1,2]. Rabies affects the host’s central nervous system [3,4] and is almost always fatal once clinical symptoms appear [1–3]. Dogs are the main rabies reservoirs in urban areas; about 20,000 to 60,000 people die each year from rabies transmitted through dog bites globally [2,5,6]. Global prevention efforts have focused on reducing the incidence of dog rabies using mass vaccination strategies for dogs living in urban and rural areas, significantly reducing the risk of human exposure to the virus [1–3,7–11].

In Mexico, the national strategy for the control and elimination of rabies, including mass dog rabies vaccination, accomplished a progressive reduction in dog rabies cases and dog-mediated human rabies deaths since the program was implemented in 1990 [8,12,13]. This elimination of rabies cases was mostly achieved through annual dog rabies vaccination campaigns. In 2019, the World Health Organization validated Mexico as the first country to eliminate rabies as a public health program [14]. Wildlife is currently considered the primary reservoir of rabies. Annual campaigns have expanded from 7.1 million dog vaccine doses administered in 1990 to 18.4 million dog vaccine doses administered in 2015, achieving estimated immunization levels of more than 80% of the dog population in most states [8,15,16]. The absence of dog-mediated human rabies deaths has been one of the critical criteria to keep funding dog rabies vaccination programs. However, no cost-effectiveness evaluation of annual dog rabies vaccination campaigns in Mexico has been performed to date.

The systematic evaluation of public health programs and interventions is essential to evaluate their continuity, redefine priorities, characterize and understand population health progress, and inform public health decision-making [17–19]. This article aims to evaluate the economic costs, effectiveness, and cost-effectiveness of the national program of rabies control in Mexico through the mass annual dog rabies vaccination campaigns, 1990–2015, from the government’s perspective.

## Methods

### Ethics statement

CENAPRECE prepared the datasets requested for the analysis. Administrative data were free of personal identifiers; all other data were publicly available in government open data repositories or published in scientific journals. The work team did not have access to sensitive information that could result in the identification of individuals.

### Methods overview

We retrospectively evaluated the national program of rabies control in Mexico, including mass annual dog rabies vaccination campaigns and post-exposure prophylaxis (PEP) for dog bite victims, from 1990 through 2015. We drew from several data sources, including administrative records from national annual dog rabies vaccination campaigns, national health and zoonosis statistics, and scientific literature. We compared the current scenario of a national program of rabies control to a counterfactual scenario of what would have happened if the Ministry of Health in Mexico had not implemented mass dog-vaccination campaigns but had instead offered PEP to dog bite victims. We used *RabiesEcon*, a mathematical modeling tool developed by the US Centers for Disease Control and Prevention (CDC) [20,21], to estimate the counterfactual scenario without mass dog-vaccinations. We used epidemiological data of dog rabies in Mexico corresponding to the year of initiation of the mass vaccination campaigns of dogs against rabies, 1990, as our baseline estimate, and estimated the total cases of human and dog rabies from 1990 through 2015. We provide further details below.

### Transmission model: *RabiesEcon*

We estimated the counterfactual scenario using *RabiesEcon* [20,21]. This tool is an adaptation of the deterministic mathematical model developed by Zinsstag et al.[22] on dynamics of rabies transmission in dogs and humans. *RabiesEcon* can estimate cases of rabies transmission and the cost of death averted and years of life gained from vaccination programs. This tool has been adapted and used in several low and middle-income countries with a potential risk of transmission of canine rabies [20], including Mexico, Tanzania, and Zambia, showing reliable results. *RabiesEcon* can estimate the potential cases of rabies in dogs and humans in different vaccination scenarios [20,21]. The main assumptions of our evaluation include (a) canine rabies is endemic in the no vaccination scenario (i.e., it is stable); (b) mass vaccination programs are implemented in a 10-week time interval; (c) the dog population can only increase up to 120% beyond the size of the population entered initially into the model; (d) the design and implementation of vaccination and control programs for animals with rabies are applied in the same way in urban and peri-urban areas (a complete list of assumptions of our evaluation is included in the Supporting Information S1 Text; also see Borse et al.[20] and Jeon et al.[21] for other applications).

Considering that the counterfactual scenario of no annual mass dog rabies vaccination campaign results in a higher proportion of rabid dogs in the country, we assumed that the proportion of dog bites that would have resulted in a bite investigation would remain equivalent to 1990 (the first year of mass vaccination campaign). We used the total number of bite victims from suspected rabid dogs to estimate the number of dogs in isolation and quarantine. We assumed that all such rabies exposures would have resulted in a laboratory investigation. We examined the validity of these assumptions using an extensive sensitivity analysis.

### Epidemiology and demography

Table 1 shows the main parameters used to estimate the counterfactual scenario without mass dog rabies vaccination, using *RabiesEcon* [20].

**Table 1:** Primary demographic and epidemiological data used to define the counterfactual scenario without a national dog rabies vaccination campaign, 1990, using *RabiesEcon* [20].

Input	Model values	Source
**Epidemiology and demography**		
Area of implementation^a^ (km^2^)	171,817	SEDATU
Human population^b^	58,407,633	INEGI
Human population density (per km^2^)	339.9	Calculated
Human birth rate (per 1000 population)^b^	27.9	INEGI
Human life expectancy^b^	70.4	INEGI
Number of humans-per-dog	6.5	Wallace et al.[7]
Dog population	8,917,713	Calculated
Dog population density (per km^2^)	51.9	Calculated
Dog birth rate (per 1000 population)^c^	350	Tlaxcala
Dog life expectancy^d^	3.20	Tlaxcala
Dog-to-dog bites from suspected rabid dogs^e^	2.35	CENAPRECE
Rabies R_o_ dog-to-dog	1.14	Calculated
Annual deaths in the program	276	Calculated
**National program of rabies control**		
Proportion of the dog population that is vaccinated^f^	0.0%	SINAIS
Vaccination frequency	Anual	Model assumption
Proportion of spayed or neutered dogs	0.0%	Model assumption
Probability of receiving PEP^g^	30.1%	SINAIS
**Epidemiology**		
PEP efficacy^h^	0.90	CENAPRECE
Dog rabies vaccine efficacy^i^	0.95	Manning et al.[23]
Probability of acquiring rabies if exposed with no PEP	0.19	Shim et al.[24]
Share of bite victims who do not seek medical care	20.5%	CENAPRECE
Share of bite victimos who receive PEP without dog vaccination program	30.1%	SINAIS
**Costs (MXN pesos 2015)**		
Isolation and/or quarantine ^j^	350.3	CENAPRECE
Laboratory testing of dogs	251.5	CENAPRECE
Bite investigation	36.7	CENAPRECE
Cost / vaccine^k^	variable	Calculated
Cost / PEP^k^	variable	Calculated

Notes.

^a^ Territorial extension of urban and peri-urban areas as reported by the Secretaría de Desarrollo Agrario, Territorial y Urbano (SEDATU).

^b^ Reported by the Instituto Nacional de Información y Estadística (INEGI) [25].

^c^ Estimated using 2014–2015 data from rabies control in the State of Tlaxcala for dogs younger than one year of age.

^d^ Estimated using 2014–2015 data from rabies control in the State of Tlaxcala for dogs older or equal to one year of age.

^e^ Estimated average 2007–2015 (supporting information S1). CENAPRECE: Centro Nacional de Programas Preventivos y Control de Enfermedades

^f^ The Dirección General de Información en Salud, Sistema Nacional de Información en Salud SINAIS [26] estimates vaccination rate is 69.4% based on state health services. We used 0% vaccination for our counterfactual scenarios with no vaccination program.

^g^ Estimated from cases with a high risk of exposure to rabies reported to SINAIS [26]. Data are an estimate of state health services.

^h^ The proportion of dog bite victims who do not finish PEP was estimated from a retrospective review of clinical records of people with suspected rabies cases.

^i^ Vaccine efficacy is estimated at 95%, provided that bite victims follow the recommended dosage schedule [23].

^j^ Estimated value of the quarantine, considering four site visits per dog, with an estimated average time of four hours per visit.

^k^ Cost per vaccine and complete PEP regimen varies annually, as estimated by CENAPRECE (S1 Text).

We combined several data sources to characterize the national program of rabies control in urban areas of Mexico (1990–2015). We used national registries from mass rabies vaccination campaigns from 1990 to 2015; data on metropolitan areas and population reported by the Secretaría de Desarrollo Agrario, Territorial y Urbano and the Instituto Nacional de Información y Estadística (INEGI) [25,26]. Dog birth rates were estimated by the Centro Nacional de Programas Preventivos y Control de Enfermedades (CENAPRECE), and defined as the number of births per year per 1,000 dogs. To estimate dog life expectancy and project dog population, we used age ranges registered in the dog rabies vaccination certificates from a convenience sample of six municipalities in the State of Tlaxcala in 2013–2016. A more detailed description of the epidemiological and demographic data used for the program’s retrospective evaluation is provided in S1 Text.

We estimated years of potential life lost (YLL) due to premature death from rabies infection using life expectancy in Mexico [26] and the distribution of suspected human exposures to rabies by age [27] (Table C in S1 Text). We obtained the number of rabid dogs from data reported by CENAPRECE. We analyzed 97 epidemiological studies carried out by state health services to estimate the average number of dogs attacked by a rabid dog, complemented with a study of the distribution of dog rabies cases in Hermosillo, México [27]. The number of people exposed to rabies from dog bites and the number of people who received PEP were estimated from national records provided by the CENAPRECE and an epidemiological study of dog bites in Hermosillo, México [26,27]. To estimate human rabies exposures, we used the probability of acquiring rabies if the bite victim was exposed to rabies but did not receive PEP [24] and the proportion of bite victims in Mexico who seek healthcare (from the Ministry of Health). To estimate dog rabies’ cases, we used the average number of attacks on humans by a rabid dog, as reported by the Ministry of Health in Mexico. A more detailed description of data sources, calculations, and main assumptions is shown in the S1 Text.

### Programmatic and economic variables

We used administrative records to estimate programmatic variables: annual dog vaccination coverage, cost per dog vaccinated, post-exposure prophylaxis (PEP), bite investigations, laboratory tests, and the number of dogs quarantined or put in isolation [20,23,24,28,29]. We also estimated the costs and benefits of the mass dog vaccination campaign in urban areas from the government’s perspective [30]. Specifically, we estimated the number of dog-mediated human rabies deaths averted, dog rabies averted, and the national vaccination program’s total costs. All monetary values presented are in 2015 Mexican pesos (MXN).

### Sensitivity analysis

We carried out a sensitivity analysis, focusing on two fundamental aspects: the variables with more significant uncertainty and the variables that could change the model’s final results or affect a health policy recommendation. The principal sensitivity analysis considered two counterfactual scenarios: low and high dog-to-dog rabies transmission [28]. We also defined two additional scenarios with more conservative and less conservative assumptions, compared to our reference scenario, about the number of investigations per dog bite, laboratory diagnoses, and people receiving PEP (further details in S1 Text).

## Results

**Fig 1:**
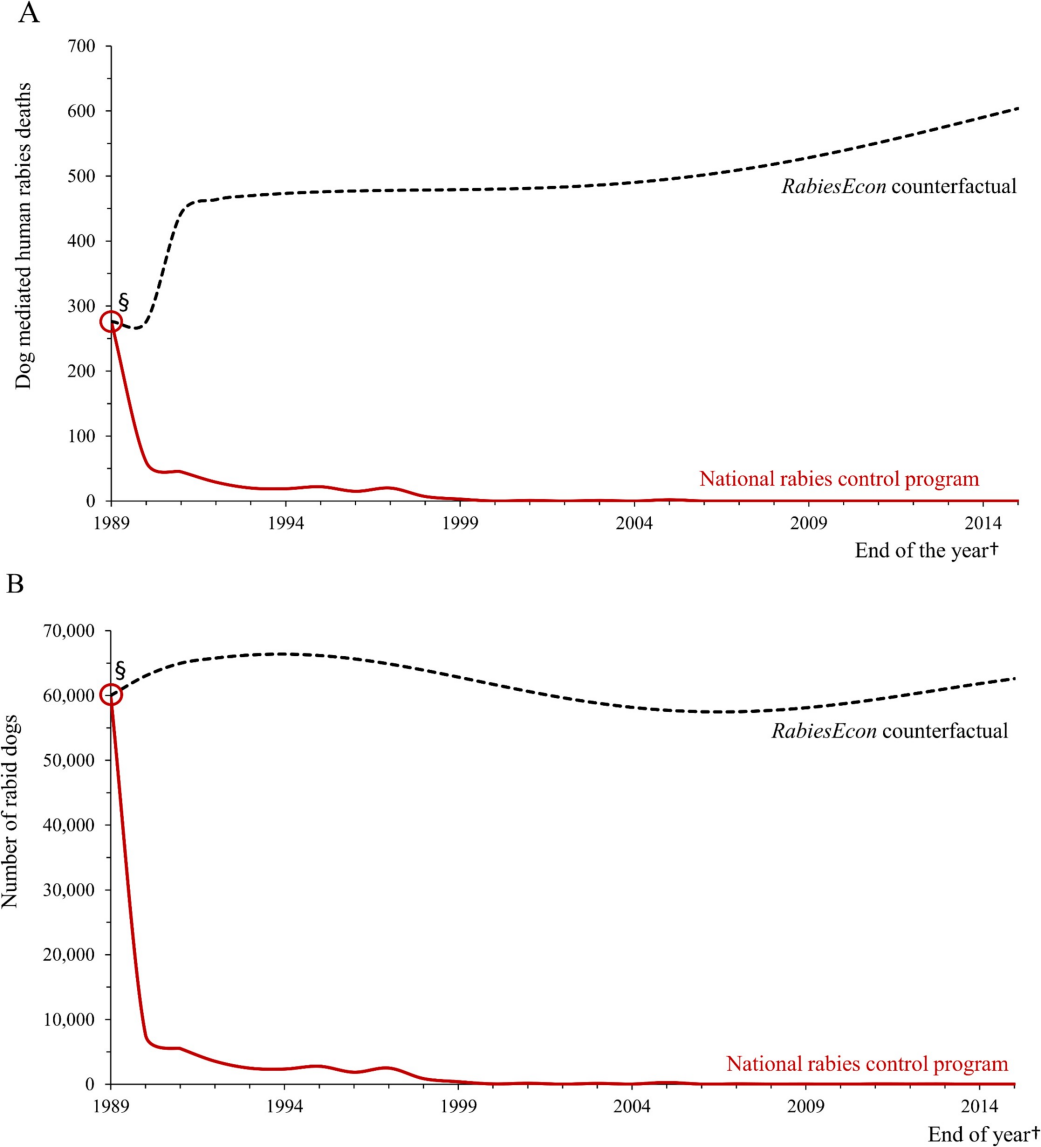
Estimated annual results of the national program of rabies control in Mexico 1990–2015: Dog mediated human rabies deaths with the program in place, as reported by CENAPRECE, and without the program, as estimated by *RabiesEcon* (A); and estimated number of rabid dogs with the program, as reported by CENAPRECE, and without the program, as estimated by *RabiesEcon* (counterfactual) (B). § For the counterfactual scenario (no dog vaccination), we estimated the annual number of dog-mediated human deaths and dog rabies cases using the population and rabies transmission parameters reported by CENAPRECE and modeled using *RabiesEcon*.

Fig 1 and Table 2 show dog mediated human deaths from dog rabies and the estimated number of rabid dogs for the two comparison scenarios: current scenario based on observed data and the counterfactual scenario estimated with *RabiesEcon* (estimates for a third scenario, with no mass dog rabies vaccination campaigns and without the availability of PEP, are shown in S1 Text). The first national dog vaccination campaign as part of the national program of rabies control in Mexico occurred in 1990. Reported data correspond to the end-of-the-year estimates; thus, the estimated annual rabies cases in 1990 are after vaccination. Table 2 also shows health results as estimated years of life lost due to premature death. Both results suggest a robust difference in the scenarios with and without an annual mass dog rabies vaccination program.

**Table 2:** Main estimated health indicators for the national program of rabies control in Mexico 1990–2015: dog-mediated human rabies deaths, years of life lost (YLL), and number of rabid dogs.

Year	Rabies deaths without annual mass dog rabies vaccination campaigns (*RabiesEcon*)			Rabies deaths with annual mass dog rabies vaccination campaigns (current scenario)		
	Human	YLL^a^	Rabid dogs	Human	YLL^a^	Rabid dogs
1990	276	14 403	63 026	60	3 131	7 652
1991	448	23 555	64 965	45	2 367	5 529
1992	476	25 210	65 766	29	1 537	3 588
1993	488	26 049	66 246	20	1 067	2 458
1994	497	26 687	66 380	19	1 019	2 341
1995	504	27 176	66 174	22	1 187	2 748
1996	508	27 525	65 656	15	813	1 869
1997	510	27 742	64 881	20	1 089	2 542
1998	510	27 850	63 917	7	382	906
1999	508	27 879	62 839	3	164	429
2000	507	27 862	61 726	0	0	48
2001	504	27 829	60 647	1	55	146
2002	503	27 813	59 664	0	0	49
2003	502	27 840	58 827	1	55	133
2004	503	27 934	58 172	0	0	5
2005	505	28 116	57 726	2	111	251
2006	509	28 399	57 499	0	0	26
2007	515	28 792	57 494	0	0	0
2008	523	29 198	57 702	0	0	1
2009	533	29 730	58 105	0	0	3
2010	545	30 399	58 676	0	0	1
2011	559	31 248	59 379	0	0	52
2012	574	32 164	60 172	0	0	38
2013	590	33 194	61 007	0	0	48
2014	606	34 251	61 833	0	0	9
2015	623	35 304	62 598	0	0	4
**Total**	13 327	734 149	1 601 076	244	12 978	30 874

**Notes.** Results were derived by combining CENAPRECE data and modeling using *RabiesEcon* [20], adapted to the annual dog vaccination campaign in Mexico by the CENAPRECE team. On average, there were 58 reported annual deaths before 1990, with substantial annual variation. While there was no national concerted and coordinated dog vaccination campaigns before 1990, some dogs were vaccinated against rabies, and about 30% of humans exposed to rabies received PEP. We used 58 deaths to initiate *RabiesEcon*, as a benchmark for circulating rabies virus in Mexico. Based on the factors that affect rabies virus transmission (Table 1), we estimated 276 human deaths in the counter factual scenario of what would have occurred without any dog vaccinations. We estimated human rabies exposures based on the probability of acquiring rabies if the bite victim was exposed to rabies but did not receive PEP [24] and the proportion of bite victims in Mexico who seek healthcare (also see [31]).

^a^ YLL: years of life lost from premature death. YLLs were estimated based on the life expectancy in Mexico, and the estimate of dogs with rabies was obtained from data reported by CENAPRECE. Table J in the S1 Text shows additional results for a scenario with no mass dog rabies vaccination campaigns and without the availability of PEP for bite victims.

The costs associated with rabies included costs of the vaccination campaign, PEP for bite victims, community bite investigations, quarantine and isolation of suspected rabid dogs, and costs of laboratory investigations. The estimated average cost per dose of dog rabies vaccine was approximately $8.44, and the average cost per vaccinated dog was $14.16 (Tables E and F, respectively, in S1 Text). This estimate considered the costs of health supplies used in the implementation of the campaign, such as syringes, vaccines, ice, biological transport thermos, vaccination certificates, and soap, personnel costs, such as salaries for vaccinators, brigade supervisors, coordinators, and non-medical costs, such as gasoline, and workers’ per diem. We estimated that about 80% of people who had been bitten by a dog seek healthcare treatment; of those, approximately 24% receive PEP, of whom about 11% do not finish the treatment (Table D in S1 Text). The average cost of PEP was estimated at approximately $643.00. Details of vaccination campaign costs, PEP for bite victims, and cost estimates are shown in more detail in S1 Text.

Table 3 shows the main results for the average cost-effectiveness of the national program of rabies control in Mexico between 1990 and 2015, from the government’s perspective. The results suggest that the program has prevented approximately a total of 13,000 human deaths from rabies, equivalent to 721,000 years of life lost due to premature death averted. The economic costs associated with the program amount to approximately $ 4,700 million Mexican pesos (USD 300 million). The program has saved about $200 million in bite research, dog quarantine and isolation, laboratory, and PEP from a decrease in rabies incidence. The average cost for averted dog-mediated human rabies death was approximately $360,000 (USD 23,000), and $6,500 (USD 410) per year of life gained. Finally, the cost per averted dog rabies case was approximately $3,000 (USD 190).

**Table 3:** Main results for the average cost-effectiveness evaluation of the national program of rabies control in Mexico, 1990–2015 (MXN 2015), compared with an estimated counterfactual scenario without mass dog rabies vaccination program, from the government’s perspective.

Indicator	Without dog vaccination program (counterfactual)	With dog vaccination program (current situation)	Difference
**Epidemiologic**			
Total dog rabies cases	1 601 076	30 854	1 570 089
Dog mediated human rabies deaths	13 327	244	13 083
Years of life lost	734 149	12 978	721 171
**Costs (MXN peso)**			
Dog vaccination campaign	-	4 836 123 729	4 836 123 729
Dog bite investigations^a^	41 928 504	35 295 083	-6 633 421
Dog isolation and quarantines^a^	529 622 122	497 852 480	-31 769 642
Laboratory investigations^a^	238 669 607	215 863 562	-22 806 045
PEP^b^	600 695 835	494 560 020	-106 135 815
Total	1 410 916 067	6 079 694 874	4 668 778 80
**Average cost-effectiveness 1990–2015**^**c**^			
Cost per dog rabies case averted	-	2 973	2 973
Cost per human death averted	-	356 865	356 865
Cost per life-year gained	-	6 474	6 474

**Notes.** The evaluation only considered dog rabies transmission and one annual dog vaccination campaign with no reinforcement. We did not consider sterilization activities. The methods and main assumptions are explained further in the discussion section and in S1 Text.

^a^ To estimate the number of dogs in isolation and quarantine and the number of laboratory investigations, the additional number of rabies exposures were estimated using *RabiesEcon* [20]. We assumed that all rabid dog bites to humans would have been investigated in a setting without dogs’ mass vaccination (see sensitivity analysis for further details).

^b^ The proportion of people bitten by a suspected rabid dog who begin PEP is higher with more endemic rabies transmission. In contrast, in scenarios with lower rabies transmission, the proportion of people receiving PEP would also decrease. For the scenario without mass vaccination, we used PEP treatment initiation rates of 1990 (i.e., at the beginning of the mass vaccination campaigns).

^c^ Average cost-effectiveness included vaccination, PEP, dog bite investigation, dog isolation and quarantines, and laboratory investigations. Using the 2015 exchange rate of USD 1 = 15.70 MXN, the average cost per dog rabies cases averted was USD189, cost per human death averted was USD 22 737, and the cost per life-year gained was USD 412. The gross domestic product per capita in Mexico in 2015 was USD 12 041 [32]. Because death is the only clinical outcome in most rabies cases, a disability-adjusted life year saved is equivalent to a year of life gained.

The results from the evaluation of the national program of rabies control in Mexico depends on the definition of the counterfactual scenario, that is, what would have happened without a mass dog vaccination program in Mexico. Table 4 shows the main results for two additional counterfactual scenarios: a low rate of dog-to-dog rabies transmission and a high rate of dog-to-dog rabies transmission (specific parameters for each scenario are shown in more detail in S1 Text). Table 4 shows that our cost-effectiveness evaluation’s main results are robust to different assumptions about dog rabies’ epidemiology in Mexico. The dog vaccination program shows better cost-effectiveness indicators in a hypothetical counterfactual scenario of high dog-to-dog rabies transmission (R_0_ = 1.75), compared to the low dog-to-dog rabies transmission scenario (R_0_ = 1.07). In the high transmission scenario, a mass dog rabies vaccination program results in a greater number of dog-mediated human rabies death and dog rabies deaths averted. We show two additional scenarios in S1 Text. We further vary our assumptions to estimate the number of investigations per bite, laboratory tests, and people receiving PEP in a counterfactual setting. The main results shown in Table 3 were also robust to these changes; that is, they would not change any of our conclusions or public policy implications.

**Table 4:** Sensitivity analysis. Main results for the average cost-effectiveness evaluation of the national program of rabies control in Mexico, 1990–2015 (MXN 2015), in two hypothetical scenarios of (i) low dog-to-dog rabies transmission (R_0_ = 1.07), and (ii) high dog-to-dog rabies transmission (R_0_ = 1.75)^a^.

Indicator	Without dog vaccination program (counterfactual)	With dog vaccination program (current situation)	Difference
**i. Low rabies transmission (R0 = 1.07)**			
**Epidemiologic**			
Total dog rabies cases	1 270 486	25 432	1 245 054
Dog mediated human rabies deaths	12 912	244	12 668
Years of life lost	711 408	12 978	698 430
**Costs (MXN peso)**			
Dog vaccination campaign	-	4 836 123 729	4 836 123 729
Dog bite investigations	41 928 504	35 295 083	-6 633 421
Dog isolation and quarantines	528 623 285	497 852 480	-30 770 805
Laboratory investigations	237 952 585	215 863 562	-22 089 023
PEP	600 695 835	494 560 020	-106 135 815
Total	1 409 200 209	6 079 694 874	4 670 494 666
**Average cost-effectiveness 1990–2015**			
Cost per dog rabies case averted		3 751	3 751
Cost per human death averted		368 671	368 671
Cost per life-year gained		6 687	6 687
**ii. High rabies transmission (R0 = 1.75)**			
**Epidemiologic**			
Total dog rabies cases	2 419 314	39 898	2 379 417
Dog mediated human rabies deaths	15 805	244	15 561
Years of life lost	871 696	12 978	858 717
**Costs (MXN peso)**			
Dog vaccination campaign	-	4 836 123 729	4 836 123 729
Dog bite investigations	41 928 504	35 295 083	-6 633 421
Dog isolation and quarantines	535 499 167	497 852 480	-37 646 687
Laboratory investigations	242 888 482	215 863 562	-27 024 920
PEP	600 695 835	494 560 020	-106 135 815
Total	1 421 011 988	6 079 694 874	4 658 682 887
**Average cost-effectiveness 1990–2015**			
Cost per dog rabies case averted	-	1 958	1 958
Cost per human death averted	-	299 373	299 373
Cost per life-year gained	-	5 425	5 425

**Notes.** The same notes as in Table 3 apply.

^a^ The parameters for the sensitivity analysis are in S1 Text.

## Discussion

Mexico has continuously included human rabies prevention into its federal public health programs and has invested substantial resources to prevent and treat possible dog rabies exposures [5,12,13,33]. The National Rabies Control Program has virtually eliminated dog mediated human rabies deaths and dog rabies cases in the past 25 years. From 2006 through 2012, no dog mediated human rabies cases were reported, and reported rabid dogs decreased from 42 in 2007 to 12 in 2012 [5,26,33]. Consistent with evidence from vaccination studies in other countries [5,20,22], this reduction was achieved primarily through annual mass dog rabies vaccination campaigns, with more than 80% of the dog population immunized in most states [8,15].

We comprehensively analyzed the cost-effectiveness of mass vaccination campaigns for dogs in Mexico. Our study is aligned with global health agencies’ recommendations to evaluate the impact of health programs and interventions to support decision-making [17,18]. Although annual mass dog vaccination campaigns are expensive, the cost per additional year of life gained is well below the threshold for interventions considered highly cost-effective according to the recommendations of the World Health Organization’s Commission on Macroeconomics and Health [34]. These thresholds classify interventions as cost-effective if the costs to avert a disability-adjusted life-year is less than one (very cost-effective) or three (cost-effective) times the country’s gross domestic product per capita. The thresholds are intended as a reference only and should not be used as a unique decision criterion [35–37]. Mexico’s gross domestic product per capita in 2015 was $188,955 (USD12,041) [32], so a highly cost-effective intervention would cost less than $188,955 per life-year gained. The average cost per life-year gained in rabies was $6500 (USD 412) and $357,000 per life saved (USD 22,737). This estimate represents a higher cost per life saved compared to a similar program (with only 50% of dogs vaccinated) in East Africa (USD 385–451) [20], and higher than a similar program in Chad (USD 596 per human death averted) [22]. Mindekem et al. reported a lower cost of USD 121 per life-year gained in Chad [38], lower than our estimated average of USD 412 for Mexico. These differences probably reflect the relatively higher costs of implementing rabies control and prevention interventions in a middle high-income country.

The study’s main results are robust to changes in our main assumptions to generate the counterfactual comparison scenario (estimated with *RabiesEcon* [20]). The conclusions and implications for public health did not change when using counterfactual scenarios of low or high transmission of rabies between dogs, nor using more and less conservative assumptions for the number of investigations of dog bites, quarantine and isolation of dogs, the number of laboratory tests, and the percentage of people receiving PEP (S1 Text). We also included a scenario with no public health interventions (i.e., no mass dog rabies vaccination or PEP for dog bite victims) in S1 Text.

The results shown suggest that PEP treatment is highly effective in saving lives in people who have been attacked by rabid dogs. Consistent with studies in countries with endemic rabies transmission, our results show that PEP treatment combined with mass rabies vaccination campaigns considerably reduces human mortality from rabies, years of life lost due to premature death, and the costs of dealing with dog bite wounds [24,29,39,40]. Some authors have suggested strategies to lower PEP implementation costs, for example, by reducing the indiscriminate application of PEP treatment to people with very low or no risk of contracting rabies, based on the health of the attacking dog and the specific conditions of the incident [2,24,31].

The Ministry of Health has implemented mass dog rabies vaccination campaigns continuously since 1990. The government has kept a reliable administrative record of reported rabies cases and deaths, with a uniform reporting protocol, which allows for a robust estimate of the epidemiological situation of dog rabies in Mexico. As in other countries [19,41–44], some cases of human and dog rabies may not have been recognized or reported to health authorities, especially in the first years of the vaccination campaign. However, incomplete information about rabies becomes more unlikely as transmission decreases. An essential element to consider in reducing dog rabies is the biological used in vaccination campaigns. Mexico uses biologicals based on cell culture, which have shown a higher quality, especially in their potency in international units (IU) [45], more than double what is recommended by the Expert Committee on Rabies of the World Health Organization [2].

One of the strengths of this study is the estimation of the economic costs, effectiveness, and cost-effectiveness of the vaccination program compared to a counterfactual scenario that describes a plausible scenario of what could have happened without vaccination (but with PEP), using *RabiesEcon*. In Mexico, *RabiesEcon* was adapted and standardized for use in a joint CDC effort with workers responsible for the federal and state zoonosis programs in CENAPRECE. This tool has also been used in other contexts, to estimate the cost-effectiveness of mass rabies vaccination campaigns in Africa [20] and of preventing dog rabies’ reintroduction through dog vaccination [21].

In 2019, the World Health Organization validated Mexico as the first country to eliminate rabies as a public health program [14]. Following rabies elimination, there is still a risk that rabies could be reintroduced to the country from importation of a rabid animal, incursion from an endemic area, or host shift from an enzootic rabies variant [21,46]. For example, in Mexico’s southern border, Guatemala is still considered a rabies-endemic country, and there are sporadic dog rabies cases in Belize [47]. In the United States, where dogs are no longer considered a rabies reservoir, there are about 60 annual reports of rabid dogs who have become infected from wildlife species such as bats, and dog vaccination is still required [46,48]. Dog rabies introductions or reintroductions could potentially result in endemic transmission [49,50]. Preventive dog rabies vaccination is one strategy to prevent and control rabies following elimination. The necessary dog vaccination coverage depends on the risk of rabies reintroduction, but estimates suggest it is a cost-effective intervention [21] considering World Health Organization guidelines [34]. Other complementary strategies include border control and surveillance.

This study has some limitations. First, our estimate of the counterfactual scenario was limited to Mexico’s urban and peri-urban areas, where the vast majority of rabies cases occur. *RabiesEcon* allows distinguishing between urban and rural areas and different vaccination regimes. Because our evaluation is at the national level, there is vast variation in population density, connectivity, dog ownership practices, interactions with wildlife, among other factors that may affect rabies transmission between states. To describe such variation is theoretically possible but would require many assumptions and more disaggregated administrative and survey data, which is lacking at the moment. The objective of *RabiesEcon* is to assist decision-making; therefore, we opted for a parsimonious model that reflects policy decisions and typical constraints faced by policymakers [51]. Second, available administrative data were not always representative of the country. For example, the analysis focuses on PEP treatment implementation and costs provided by the Ministry of Health, excluding data from other social security institutions. However, rabies mortality records encompass the entire health sector. Third, we had to make several assumptions about rabies’ epidemiology and government interventions to generate a plausible counterfactual scenario. It is impossible to know what would have happened without a mass dog rabies vaccination program. To verify how much these assumptions influenced our estimates, we did an extensive sensitivity analysis that suggests that the results and main conclusions were robust (Table 4, and in Tables J, K, and L in S1 Text). We did not include stochastic variations, which may be necessary for the context of a low number of rabies infections [6]. Last, our estimates are also limited by modeling decisions. *RabiesEcon* is a modeling tool for the economic evaluation of dog rabies control programs and interventions [20]. As such, it does not provide a detailed representation of virus transmission between dogs and between dogs and humans, but rather on the factors that are most likely to affect health-policy decisions. *RabiesEcon* does not consider the dog population’s spatial distribution, variations in dog ownership preferences, or human behavior, potentially affecting the estimated number of rabies cases in dogs and humans [22,52,53].

In conclusion, this systematic evaluation shows that mass rabies vaccination campaigns for dogs in Mexico have been the primary way to progressively reduce the transmission of dog-to-dog rabies and dog mediated human rabies deaths. In 2019, the World Health Organization validated Mexico for eliminating rabies as a public health problem. The program has resulted in thousands of years of life gained from averted premature deaths since 1990. PEP treatment is an effective tool for preventing human deaths in all scenarios analyzed and combined with dog vaccination are a highly cost-effective strategy to prevent deaths from rabies. Following rabies elimination, there is still a risk that rabies could be reintroduced to the country from importation, incursion, or host shift. Mass dog vaccination has been a successful strategy to progressively reduce and finally eliminate rabies transmission since the program began in 1990.

## Supporting information

S1 Text 1. Additional background; 2. Transmission model: *RabiesEcon;* 3. Model inputs; 4. Additional results: health indicators of the rabies control program; 5. Additional sensitivity analysis. **Supporting figure legends:** Figure A. Sensitivity analysis of cost-effectiveness indicators: incremental cost (with and without the dog vaccination program) for (i) dog rabies cases averted, (ii) per human death averted (in MXN 100s), (iii) per year of life gained. **Supporting table legends:** Table A. Main epidemiological and demographic variables used in the evaluation of the national rabies vaccination campaign in urban areas, Mexico 1990–2015; Table B. Vaccination certificates of the State of Tlaxcala, 2013–2016; Table C. Distribution of suspected human exposures to rabies by age; Table D. Epidemiological description of the compared scenarios: National Rabies Control Program and counterfactual scenario without annual vaccination (*RabiesEcon*); Table E. Costs associated with the dog rabies vaccine (MXN 2015); Table F. Estimated unit costs of the dog vaccine per year (MXN 2015); Table G. Treatment costs for post-exposure prophylaxis (PEP) in cases with probable dog rabies exposures (MXN 2015); Table H. Control measures for suspected rabid dogs (quarantine and isolation, lab tests, and bite investigations); Table I. Summary of costs and coverage of dog vaccination campaigns in Mexico, 1990–2015; Table J. Main health indicators of the rabies control program evaluation: human deaths from rabies, the equivalent in years of life potentially lost due to premature death (YLL), and the estimated number of rabid dogs México, 1990–2015; Table K. Sensitivity analysis: most conservative scenario. Main results for the average cost-effectiveness evaluation of the national program of rabies control in Mexico, 1990–2015 (MXN 2015), compared with an estimated counterfactual scenario without mass dog rabies vaccination program, from the government’s perspective; Table L. Sensitivity analysis: least conservative scenario. Main results for the average cost-effectiveness evaluation of the national program of rabies control in Mexico, 1990–2015 (MXN 2015), compared with an estimated counterfactual scenario without mass dog rabies vaccination program, from the government’s perspective; Table M. Parameters for the sensitivity analysis in scenarios of low and high rabies transmission, to estimate the counterfactual scenario without annual mass dog vaccination campaigns, Mexico 1990–2015.
(PDF)
