# Supplementary material for: Cost-effectiveness of the national dog rabies prevention and control program in Mexico, 1990–2015
Source: PLoS Negl Trop Dis. 2021 Mar 4;15(3):e0009130. doi: 10.1371/journal.pntd.0009130 (PMC7963054; doi:10.1371/journal.pntd.0009130)
Supplement: S1 Text — 1. Additional background; 2. Transmission model: RabiesEcon; 3. Model inputs; 4. Additional results: health indicators of the rabies control program; 5. Additional sensitivity analysis. Supporting figure legends: Figure A. Sensitivity analysis of cost-effectiveness indicators: incremental cost (with and without the dog vaccination program) for (i) dog rabies cases averted, (ii) per human death averted (in MXN 100s), (iii) per year of life gained. Supporting table legends: Table A. Main epidemiological and demographic variables used in the evaluation of the national rabies vaccination campaign in urban areas, Mexico 1990–2015; Table B. Vaccination certificates of the State of Tlaxcala, 2013–2016; Table C. Distribution of suspected human exposures to rabies by age; Table D. Epidemiological description of the compared scenarios: National Rabies Control Program and counterfactual scenario without annual vaccination (RabiesEcon); Table E. Costs associated with the dog rabies vaccine (MXN 2015); Table F. Estimated unit costs of the dog vaccine per year (MXN 2015); Table G. Treatment costs for post-exposure prophylaxis (PEP) in cases with probable dog rabies exposures (MXN 2015); Table H. Control measures for suspected rabid dogs (quarantine and isolation, lab tests, and bite investigations); Table I. Summary of costs and coverage of dog vaccination campaigns in Mexico, 1990–2015; Table J. Main health indicators of the rabies control program evaluation: human deaths from rabies, the equivalent in years of life potentially lost due to premature death (YLL), and the estimated number of rabid dogs México, 1990–2015; Table K. Sensitivity analysis: most conservative scenario. Main results for the average cost-effectiveness evaluation of the national program of rabies control in Mexico, 1990–2015 (MXN 2015), compared with an estimated counterfactual scenario without mass dog rabies vaccination program, from the government’s perspective; Table L. Sensitivity analysis: least conse [file pntd.0009130.s001.pdf]

PLoS Neglected Tropical Diseases

Supporting information

Cost-effectiveness of the national dog rabies prevention and control program in Mexico, 1990-2015

Jesús Felipe González-Roldán, Eduardo A. Undurraga*, Martin I. Meltzer, Charisma Atkins, Fernando Vargas-Pino, Verónica Gutiérrez-Cedillo, José Ramón Hernández Pérez

Contents

1. Additional background 2
2. Transmission model: *RabiesEcon* 2
   2.1 Main assumptions 3
   2.2 Economic model 3
3. Model inputs 4
   3.1 Dog birth rate and life expectancy 5
   3.2 Dog to dog bites 5
   3.3 Humans exposed to rabies 5
   3.4 Epidemiological and programmatic characterization of the National Rabies Control Program compared to counterfactual scenario without mass dog rabies vaccination (*RabiesEcon*) 7
   3.5 Economic characterization of the National Rabies Control Program compared to the counterfactual scenario without mass rabies dog vaccination (*RabiesEcon*) 9
4. Additional results: health indicators of the rabies control program 14
5. Additional sensitivity analysis 15
   5.1 Alternative scenarios for public health services complementary to dog vaccination 15
   5.2 Alternative scenarios for rabies transmission 19
6. References in supplementary material 20

*eundurra@uc.cl

1. Additional background

The national strategy for controlling and eliminating rabies, including mass dog rabies vaccination, accomplished a progressive reduction in dog rabies cases and dog-mediated human rabies deaths since the program began in 1990 [1-3]. The government of Mexico achieved this elimination of rabies cases through annual dog rabies vaccination campaigns. In 2019, the World Health Organization validated Mexico as the first country to eliminate rabies as a public health program [4]. The national strategy coincides with the Pan American Health Organization's initiative to eliminate urban dog rabies in Latin America [2,3]. This strategy began in 1990-1994 under the National Health Program and continued in subsequent federal government administrations (1995-2000; 2001-2006, 2007-2012 and 2013-2018).

From 1990-1999, 31 states reported rabies cases. Baja California was the only state with no dog rabies cases. Colima, Nuevo León, Quintana Roo, and Tamaulipas were classified as in "low enzootic transmission transition zones", with sporadic notification and less than ten reported cases in 1990-1999. Those states reported 0.16% of all cases. States classified as low enzootic transmission zones reported 1.7% of rabies cases, and included Aguascalientes, Baja California Sur, Campeche, Chihuahua, Nayarit, Sinaloa, Tabasco. These states had between 11 and 120 rabies cases reported. States with medium and high enzootic transmission represented 98% of dog rabies cases and included Chiapas, Coahuila, Federal District, Durango, Guanajuato, Guerrero, Hidalgo, Jalisco, Edo. Mexico, Michoacán, Morelos, Oaxaca, Puebla, Querétaro, San Luis Potosí, Sonora, Tlaxcala, Veracruz, Yucatán, and Zacatecas.

In 2000-2009 20 states reported dog rabies cases. Twelve states reported no cases, including Aguascalientes, Baja California, Baja California Sur, Colima, Guanajuato, Jalisco, Nayarit, Nuevo León, Querétaro, Quintana Roo, San Luis Potosí and Tabasco. Low enzootic transmission transition zones accounted for 3.8% of cases and included nine states: Campeche, Chihuahua, Federal District, Michoacán, Morelos, Sinaloa, Sonora, Tamaulipas, and Zacatecas. Low enzootic transmission zones included nine states that reported 31.9% of the total cases: Chiapas, Coahuila, Durango, Guerrero, Hidalgo, Oaxaca, Tlaxcala, Veracruz, and Yucatán. The states of Mexico and Puebla were considered medium and high enzootic transmission zones, accounting for 64.2% of the total reported dog rabies cases.

In 2010-2015, only 11 states reported cases. 21 states reported no rabies cases, including Aguascalientes, Baja California, Campeche, Chihuahua, Coahuila, Colima, Durango, Guanajuato, Guerrero, Hidalgo, Morelos, Nayarit, Oaxaca, Quintana Roo, Sinaloa, Sonora, Tabasco, Tamaulipas, Tlaxcala, Veracruz, and Zacatecas. Nine states accounted for 18.6% of cases, and were classified as transition zones of low enzootic transmission: Baja California Sur, Federal District, Jalisco, Michoacán, Nuevo León, Puebla, Querétaro, San Luis Potosí, and Yucatán. The zone classified as low enzootic transmission accounted for 81.4% of cases, and included the states of Chiapas and Estado de México. The zone of medium and high enzootic transmission disappeared.

2. Transmission model: *RabiesEcon*

RabiesEcon [5] adapted the deterministic model of dog rabies transmission of Zinsstag et al. [6], which includes dog-dog and dog-human transmission. The introduction of the rabies virus into a susceptible population initially causes a substantial variation in the estimation of weekly cases of canine rabies. *RabiesEcon* uses demographic and epidemiological data entered by the user and assumes that rabies' introduction occurs by a single dog that initiates transmission in the dog population. The baseline model, that is, the scenario without vaccination, shows cases of rabies in dogs and in humans once the transmission model stabilizes and reaches equilibrium.

2.1 Main assumptions

i. Mexico's annual rabies vaccination campaign considers dogs and cats. Our evaluation considered only rabies transmission in dogs since they represent the primary transmitter of rabies.

ii. The rabies vaccination campaign in Mexico includes a mass national dog vaccination campaign (March) and a subsequent reinforcement (September); we assumed all epidemiological and cost data represents both campaigns.

iii. Considering that rabies is a notifiable disease, the model assumes no underreporting of rabies in humans throughout the study period.

iv. The costs per dog vaccinated were based on the 2015 budget in Mexico. The costs corresponding to basic campaign resources were considered constant over time, while the biological variation varied during the analysis period. All cost data were adjusted to 2015 pesos.

v. We assumed a constant urban perimeter throughout the study and uniform spatial distribution of the dog population. *RabiesEcon* assumes density-dependent transmission [5,6]. Some research suggests that infectious contacts may be independent of dog density [7].

vi. The calculation of rabies cases without mass dog vaccination (counterfactual scenario) was initiated with 58 dog-mediated human rabies deaths, which corresponds to the average number of human rabies deaths in 1970-1989 in Mexico.

vii. Actions related to sterilization were not considered as part of the analysis.

viii. To estimate the probability that an offending dog is put in quarantine, we used the estimated proportion of dog attacks at home in Mexico. The government's rabies control program includes bite investigations, and dogs with a known owner are typically put in quarantine.

ix. Considering that there is probably a higher percentage of rabid dogs in the no dog vaccination scenario, we considered that the number of dog bite investigations would remain the same as in 1990. 1990 is the year in which the mass dog vaccination campaigns began. To estimate the number of dogs put in isolation and quarantine and the number of dog laboratory investigations, we added the additional number of rabies exposures estimated with *RabiesEcon* [5], assuming that all bites from rabid dogs would have been investigated in the scenario without mass dog vaccination.

x. As observed in rabies vaccination campaigns in Latin America, we assumed that the percentage of the population that begins rabies treatment following a dog bite is higher with more dog rabies transmission. With less rabies circulating, the proportion of people receiving post-exposure prophylaxis (PEP) treatment also decreases. We used the preventive treatment initiation rate of 1990 (i.e., at the beginning of the mass vaccination campaigns) for no vaccination scenario.

2.2 Economic model

RabiesEcon evaluates the costs associated with suspected rabies exposures (i.e., cost of bite investigation, laboratory diagnosis, animal isolation and or quarantine, vaccination, and PEP) and the costs associated with the control of the dog population (i.e., sterilization) and vaccination campaigns. The model's specific variables, calculation methods, and the *RabiesEcon* tool for users are available in Borse et al.[5] and Jeon et al.[8].

3. Model inputs

Table A. Main epidemiological and demographic variables used in the evaluation of the national rabies vaccination campaign in urban areas, Mexico 1990-2015

		Humans			Dogs				
**Year**	**Urban area (km^2)[a]**	**Population[b]**	**Birth rate /1000[b]**	**Life expectancy[b]**	**Population**	**Birth rate /1000[c]**	**Life expect[d]**	**Dog-dog bites[e]**	**Humans: dogs**
1990	171,817	58,407,633	27.91	70.4	8,917,713	442	3.0	2.35	6.5
1991	171,818	59,942,314	27.54	70.8	9,618,246	442	3.0	2.35	6.2
1992	171,819	61,524,160	27.15	71.2	10,046,855	442	3.0	2.35	6.1
1993	171,820	63,154,782	26.75	71.6	10,723,006	442	3.0	2.35	5.9
1994	171,821	64,835,855	26.34	71.9	11,136,010	442	3.0	2.35	5.8
1995	171,822	66,561,905	25.87	72.2	11,540,121	442	3.04	2.33	5.8
1996	171,823	67,818,746	25.36	72.4	11,839,808	442	3.04	2.33	5.7
1997	171,824	69,105,724	24.85	72.7	12,203,789	442	3.04	2.33	5.7
1998	171,825	70,423,682	24.34	72.9	12,570,722	442	3.04	2.33	5.6
1999	171,826	71,773,487	23.87	73.1	13,015,006	442	3.04	2.33	5.5
2000	171,827	73,154,122	23.42	73.2	13,377,540	401	3.08	2.31	5.5
2001	171,828	74,194,614	22.99	73.4	14,258,436	401	3.08	2.31	5.2
2002	171,829	75,254,281	22.58	73.5	14,734,202	401	3.08	2.31	5.1
2003	171,830	76,333,575	22.19	73.7	15,041,164	401	3.08	2.31	5.1
2004	171,831	77,432,950	21.85	73.8	15,279,467	401	3.08	2.31	5.1
2005	171,832	78,548,242	21.48	73.9	15,742,033	381	3.12	2.29	5.0
2006	171,833	80,043,460	21.13	74.0	15,979,175	381	3.12	2.29	5.0
2007	171,834	81,571,889	20.78	74.1	16,200,520	381	3.12	2.29	5.0
2008	171,835	83,134,347	20.43	74.0	16,551,604	381	3.12	2.29	5.0
2009	171,836	84,731,666	20.08	74.0	16,807,804	381	3.12	2.29	5.0
2010	171,837	86,357,931	19.71	74.0	17,023,580	370	3.16	2.27	5.1
2011	171,838	87,741,588	19.44	74.1	17,274,781	370	3.16	2.27	5.1
2012	171,839	89,149,940	19.20	74.3	17,586,013	370	3.16	2.27	5.1
2013	171,840	90,583,461	18.96	74.5	17,888,888	370	3.16	2.27	5.1
2014	171,841	92,042,631	18.74	74.7	18,072,226	370	3.16	2.27	5.1
2015	171,842	93,521,784	18.52	74.9	18,528,124	360	3.20	2.25	5.0
Source		INEGI			CENAPRECE				

Notes: [a] Territorial extension of the metropolitan areas reported by the Secretaría de Desarrollo Agrario, Territorial y Urbano (SEDATU). [b] Instituto Nacional de Información y Estadística (INEGI) [9]. [c] Estimated based on data from Tlaxcala 2014-2015 dogs <1-year-old. [d] Estimated based on data from Tlaxcala 2014-2015 dogs ≥1-year-old. [e] Average dog bites 2007-2015. [f] Estimated with *RabiesEcon* [5], and considers a risk of rabies infection (dog-dog) of 0.35.[g] Registered in the Sistema de Información en Salud [10].

3.1 Dog birth rate and life expectancy

Estimated using the crude birth rate formula:

$$b = \frac{B}{P} \times 1000$$

where:

b= Crude birth rate

B= Total number of births in a year

P= Total population

Values were obtained from dog vaccination certificates. Specifically, we used a convenience sample from 2013-2016 of 24,782 dog vaccination certificates corresponding to six municipalities and the same number of localities in Tlaxcala. The information was tabulated in an Excel sheet, where vaccination reports were recorded by age range in months for dogs less than one-year-old and then by years.

Considering only dogs under one year of age, we estimated birth rates for 2014 and 2015 of 291 and 265 per thousand, respectively. From these birth rates, the average rate and an associated confidence interval (CI) were estimated, 278.08 with an IC (113.8-442.3). For its evolution in the 25 years, we assumed that at the beginning in 1990, it would have a value close to the lower limit of the CI, and we then adjusted the hits rate every five years starting in 2000.

We used vaccination certificates in Table B to estimate the population of dogs ≥l years of age (2014-2015). The average age was 3.2 years (IC 3.13-3.21). We used 3.13 years and linearly increased dogs' life expectancy until 2015.

3.2 Dog to dog bites

The number of dogs attacked by a suspected rabid dog was estimated from a review of 97 epidemiological studies of animal rabies between 2007-2015. There were 1.56 average attacks by rabid dogs. The model required this estimate for 1990. Because there was less animal control at the beginning of the study, we assumed an initial value was 2.35 and a linear decrease of 0.02 every five years.

3.3 Humans exposed to rabies

Table C shows the distribution of human exposures to dog rabies virus in Mexico through dog aggressions and bites [11]. The distribution was used to estimate the years of life lost due to premature death from rabies.

Table B. Vaccination certificates of the State of Tlaxcala, 2013-2016

Age	2013	2014	2015	2016
<3 months	437	495	594	270
3-6 months	521	896	941	514
6 months-1year	478	802	815	725
1	563	1559	2170	971
2	457	1171	1115	694
3	362	861	748	493
4	192	537	944	329
5	173	415	803	280
6	103	246	236	163
7	60	144	127	117
8	69	151	132	104
9	29	53	57	53
10	42	97	83	59
11	10	32	44	22
12	6	36	19	35
13	11	14	10	7
14	4	6	7	8
15	3	19	15	9
16	2	0	0	3
17	1	0	2	0
18	1	1	0	1
19	0	0	1	0
20	0	1	0	2
Total	3,524	7,536	8,863	4,859

Notes. The data correspond to information from 6 municipalities in the State of Tlaxcala in a convenience sample 2013-2016.

Table C. Distribution of suspected human exposures to rabies by age.

Age group	Age equivalent	Total	Proportion (%)
**Mexico (N=312)**			
0-13	6.5	187.0	59.9%
14-19	16.5	41.0	13.1%
20+	45.2	84.0	26.9%
Total		312	

Source: Eng et al.[11].

3.4 Epidemiological and programmatic characterization of the National Rabies Control Program compared to counterfactual scenario without mass dog rabies vaccination (*RabiesEcon*)

Table D. Epidemiological description of the compared scenarios: National Rabies Control Program and counterfactual scenario without annual vaccination (*RabiesEcon*)

	Annual mass dog rabies vaccination campaigns (current scenario)						Without annual mass dog rabies vaccination campaigns (**RabiesEcon**))[d]		
Year	Reported vax coverage[a]	Prob. of post-exposure prophylaxis[b]	Adjusted PEP probability	Reported human rabies deaths	Human rabies exposures (calculated)[c]	Dog rabies cases (calculated)	Human deaths	Human rabies exposures	Dog rabies cases
1990	69%	37.9%	30.1%	60	434	7,632	276	1,993	60,000
1991	72%	33.9%	27.0%	45	316	5,559	448	3,112	63,026
1992	83%	33.4%	26.6%	29	202	3,561	476	3,291	64,965
1993	80%	33.3%	26.5%	20	139	2,439	488	3,375	65,766
1994	80%	34.7%	27.6%	19	135	2,377	497	3,483	66,246
1995	85%	35.6%	28.3%	22	158	2,782	504	3,559	66,380
1996	82%	33.5%	26.6%	15	107	1,883	508	3,516	66,174
1997	84%	36.0%	28.6%	20	143	2,510	510	3,614	65,656
1998	89%	36.0%	28.6%	7	52	915	510	3,614	64,881
1999	91%	38.5%	30.6%	3	22	391	508	3,694	63,917
2000	91%	37.0%	29.4%	0	0	2	507	3,626	62,839
2001	92%	30.3%	24.1%	1	9	165	504	3,390	61,726
2002	94%	31.7%	25.2%	0	3	57	503	3,423	60,647
2003	95%	32.4%	25.8%	1	9	160	502	3,441	59,664
2004	94%	33.8%	26.9%	0	1	20	503	3,490	58,827
2005	94%	30.7%	24.4%	2	15	259	505	3,406	58,172
2006	95%	28.2%	22.4%	0	2	34	509	3,357	57,726
2007	95%	33.2%	26.4%	0	1	25	515	3,556	57,499
2008	95%	28.3%	22.5%	0	2	34	523	3,454	57,494
2009	94%	27.1%	21.6%	0	3	52	533	3,483	57,702

Year	**Annual mass dog rabies vaccination campaigns (current scenario)**						**Without annual mass dog rabies vaccination campaigns (*RabiesEcon*))[d]**		
	Reported vax coverage[a]	Prob. of post-exposure prophylaxis[b]	Adjusted PEP probability	Reported human rabies deaths	Human rabies exposures (calculated)[c]	Dog rabies cases (calculated)	Human deaths	Human rabies exposures	Dog rabies cases
2010	93%	27.6%	22.0%	0	1	10	545	3,577	58,105
2011	96%	37.1%	29.5%	0	1	17	559	4,006	58,676
2012	93%	29.0%	23.1%	0	3	51	574	3,813	59,379
2013	92%	23.7%	18.8%	0	3	52	590	3,739	60,172
2014	94%	21.0%	16.7%	0	0	7	606	3,755	61,007
2015	90%	23.1%	18.4%	0	0	5	623	3,926	61,833
Fuente	CENAPRECE				Calculado		RabiesEcon		

Notes

[a] Data from the Dirección General de Información en Salud, Sistema de Información en Salud [10]

[b] Estimate from severe rabies exposures reported to Dirección General de Información en Salud, Sistema de Información en Salud [10].

[c] Adjusted for the estimated proportion of people exposed to rabies who do not seek healthcare. Approximately 79.5% of people bitten by dogs sought medical attention. This number was estimated from the retrospective review of 38 records of dog mediated human rabies deaths by comparing the date of the attack and the number of people attacked by a dog in that epidemiological week and in that same Municipality. To estimate human rabies exposures, we used the probability of acquiring rabies if the bite victim was exposed to rabies but did not receive PEP [12] and the proportion of bite victims in Mexico who seek healthcare (from the Ministry of Health). To estimate dog rabies' cases, we used the average number of attacks on humans by a rabid dog, as reported by the Ministry of Health in Mexico. Also, see [13].

[d] Estimated with *RabiesEcon*, using CENAPRECE defined parameters and previous literature (e.g., probability of rabies, as shown in Table 1 of the main manuscript). On average, there were 58 reported annual deaths before 1990, with a substantial annual variation. While there was no national concerted and coordinated dog vaccination campaigns before 1990, dogs were vaccinated against rabies, and about 30% of humans exposed to rabies received PEP. We used 58 deaths to initiate RabiesEcon, as a benchmark for circulating rabies virus in Mexico. Based on the factors that affect rabies virus transmission (Table 1), we estimated 276 human deaths in the counterfactual scenario of what would have occurred without any dog vaccinations.

3.5 Economic characterization of the National Rabies Control Program compared to the counterfactual scenario without mass rabies dog vaccination (*RabiesEcon*)

Table E. Costs associated with the dog rabies vaccine (MXN 2015)

Input	Cost	Unit cost	Calculation	Cost per vaccine
**Basic input**				Table F
Canine rabies vaccine bottle (20 doses)		Table F	1 dose per dog	
Syringe with needle 1 o 3 ml	$1.21	$1.21	1 syringe per application	$1.210
Vaccination certificate	$53.70	$53.70	1 certificate per vaccinated dog	$0.537
Thermos for the transport of the biological	$270.00	$270.00	1 thermos for every 12,000 doses	$0.020
Ice bag (4 kg.)	$24.00	$24.00	1 ice bag per 360 doses	$0.060
Pen	$2.84	$2.84	1 pen per 200 dog certificates	$0.010
Vaccine animal record sheet	$30.00	$0.30	1 sheet for every 100 records	$0.003
Plastic bag for wraps and syringes without needle	$30.00	$0.30	1 bag per 100 doses	$0.003
Cost per kilo of needle deposit	$12,630	$12.63	1000 needles	$0.012
Soap	$4.43	$4.43	1 soap bar per 500 doses	$0.010
Waste bag (kg)	$42.92	$42.92	1 kg per 15000 doses	$0.002
Vaccinator (cost per day)	$7,628	$254.25	Daily wage, 8:00-15:00, equivalent to an average of 60 doses	$4.230
Supervisor (cost per month)	$7,694	$256.45	Monthly salary, equivalent to about 10,000 vaccine doses administered	$0.760
Coordinator (cost per month)	$19,396	$646.53	Monthly salary, equivalent to about 100,000 vaccine doses administered	$0.190
**Subtotal basic inputs**				**$7.047**
**Complementary input**				
Gas for personnel transport (liter)	$264.00	$13.2	20 liters per vehicle to transport 10 vaccinators for 600 dog vaccinations	$0.440
Tables	$48.00	$48.0	1 Table per 6,000 records	$0.008
Per diem	$28.00	$28.0	1 per diem per worker	$0.560
Driver (month)	$7,627.70	$254.2	Monthly salary per 20,000 vax doses	$0.380
Awareness campaign	$900	$900.0	1 canvas per 100,000 vax doses	$0.010
**Sub total complementary input**				**$1.398**
**Total**			**Cost per vaccinated dog**	**$8.445**

Source: Administrative records CENAPRECE

Table F. Estimated unit costs of the dog vaccine per year (MXN 2015).

Year	Vaccine	Cost per dose	Cost per vaccinated dog[a]
1990	112.70	5.64	14.08
1991	99.54	4.98	13.42
1992	90.31	4.52	12.96
1993	87.47	4.37	12.82
1994	124.97	6.25	14.69
1995	128.10	6.40	14.85
1996	102.17	5.11	13.55
1997	90.43	4.52	12.97
1998	78.05	3.90	12.35
1999	72.30	3.61	12.06
2000	69.30	3.47	11.91
2001	66.38	3.32	11.76
2002	78.50	3.93	12.37
2003	83.53	4.18	12.62
2004	87.80	4.39	12.84
2005	86.45	4.32	12.77
2006	92.18	4.61	13.05
2007	101.28	5.06	13.51
2008	109.86	5.49	13.94
2009	143.67	7.18	15.63
2010	147.36	7.37	15.81
2011	151.09	7.55	16.00
2012	157.49	7.87	16.32
2013	190.80	9.54	17.99
2014	204.26	10.21	18.66
2015	220.00	11.00	19.45

Notes: [a]The estimate of the cost per vaccinated dog corresponds to the value of the vaccine plus supplies used in the implementation of mass dog rabies vaccination campaigns.

Table G. Treatment costs for post-exposure prophylaxis (PEP) in cases with probable dog rabies exposures (MXN 2015)

Year	Basic care	Costs				Weights by risk					PEP costs
		Mild		Severe		Basic	mild		Severe		
		Complete	Incomplete	Complete	incomplete	care	Compl	Incompl	Compl	Incompl.	
1990	36.69	383.04	262.35	2,930.17	2,809.48	62%	13%	11%	7%	7%	508.91
1991	36.69	348.12	241.40	2,529.95	2,423.22	65%	10%	13%	5%	7%	386.73
1992	36.69	328.29	229.50	2,302.75	2,203.96	67%	12%	10%	6%	5%	344.22
1993	36.69	315.98	222.11	2,161.64	2,067.77	66%	10%	12%	6%	7%	339.98
1994	36.69	305.86	216.04	2,045.62	1,955.80	67%	10%	11%	5%	6%	312.10
1995	36.69	740.47	476.81	2,802.75	2,539.09	64%	10%	13%	6%	7%	494.76
1996	36.69	771.40	495.36	3,201.99	2,925.96	66%	10%	12%	5%	6%	506.48
1997	36.69	701.89	453.66	3,637.76	3,389.52	64%	9%	13%	6%	8%	614.69
1998	36.69	815.72	521.96	3,854.03	3,560.27	63%	10%	13%	6%	8%	684.10
1999	36.69	765.64	491.91	4,020.40	3,746.67	61%	10%	15%	5%	8%	697.04
2000	36.69	907.52	577.03	4,598.43	4,267.95	65%	12%	10%	6%	6%	733.15
2001	36.69	903.74	574.77	4,576.93	4,247.96	68%	10%	11%	5%	6%	686.63
2002	36.69	1256.25	786.28	4,993.05	4,523.07	72%	8%	12%	5%	6%	724.63
2003	36.69	1262.57	790.06	4,873.46	4,400.96	66%	10%	13%	5%	6%	772.33
2004	36.69	1162.47	730.00	4,339.70	3,907.23	63%	10%	14%	5%	8%	759.00
2005	36.69	1159.79	728.40	4,138.03	3,706.63	69%	8%	11%	5%	7%	649.11
2006	36.69	1120.96	705.10	4,128.24	3,712.38	72%	8%	10%	4%	6%	587.09
2007	36.69	1373.68	856.73	4,657.02	4,140.07	68%	11%	9%	6%	5%	767.78
2008	36.69	1086.12	684.20	3,700.93	3,299.00	67%	10%	11%	6%	6%	621.07
2009	36.69	1154.77	725.38	5,083.35	4,653.97	70%	9%	10%	5%	6%	756.02
2010	36.69	1083.23	682.46	4,470.04	4,069.27	71%	9%	10%	5%	5%	626.25
2011	36.69	1169.53	734.24	4,760.97	4,325.68	61%	10%	13%	7%	9%	931.22
2012	36.69	1267.43	792.98	5,373.21	4,898.76	70%	8%	10%	5%	7%	812.04
2013	36.69	1517.55	943.05	7070.08	6495.59	77%	7%	8%	4%	4%	760.52
2014	36.69	1602.29	993.90	8983.98	8375.58	78%	6%	8%	4%	5%	948.08
2015	36.69	1322.26	825.88	8429.95	7933.57	83%	4%	6%	3%	4%	688.26

Notes. We estimated a weighted average considering the proportion of the population served in each of the scenarios, mild or severe, and considering whether PEP was complete or incomplete.
Source: Administrative records of CENAPRECE.

Table H. Control measures for suspected rabid dogs (quarantine and isolation, lab tests, and bite investigations)

Year	Reported dog bites	Prop. isolation or quarantine (%)[a]	Isolated dogs	Prob. lab tests (%)[b]	Samples studied	Prob. bite investigation (%)[c]
1990	96,922	46.0%	44,584	10.02%	9,714	37.92%
1991	91,811	46.0%	42,233	9.74%	8,938	34.70%
1992	95,999	46.0%	44,160	15.03%	14,424	33.22%
1993	81,237	46.0%	37,369	16.39%	13,312	33.89%
1994	94,297	46.0%	43,377	8.50%	8,017	33.08%
1995	87,951	46.0%	40,457	10.33%	9,086	35.59%
1996	96,698	46.0%	44,481	10.60%	10,252	33.52%
1997	96,954	46.0%	44,599	12.12%	11,750	36.01%
1998	97,851	46.0%	45,011	13.14%	12,855	36.96%
1999	107,302	46.0%	49,359	13.16%	14,123	38.51%
2000	125,686	47.0%	59,072	13.30%	16,716	34.52%
2001	138,533	47.0%	65,111	16.07%	22,265	32.17%
2002	142,641	47.0%	67,041	24.92%	35,553	30.32%
2003	147,968	47.0%	69,545	28.34%	41,929	34.34%
2004	147,335	47.0%	69,247	36.74%	54,134	36.55%
2005	145,621	47.0%	68,442	41.10%	59,850	31.14%
2006	141,574	47.0%	66,540	42.56%	60,247	28.12%
2007	123,248	47.0%	57,927	40.64%	50,087	32.09%
2008	121,071	47.0%	56,903	39.53%	47,854	32.88%
2009	113,431	47.0%	53,313	41.53%	47,105	30.00%
2010	112,677	49.0%	55,212	47.90%	53,969	28.64%
2011	119,806	49.0%	58,705	43.62%	52,264	38.81%
2012	127,525	49.0%	62,487	40.05%	51,073	29.96%
2013	112,746	49.0%	55,246	46.21%	52,096	23.08%
2014	127,084	49.0%	62,271	43.63%	55,447	22.40%
2015	119,507	49.0%	58,558	37.98%	45,383	17.37%
**Costo**		350.29[d]			251.46	36.69

Notes. Data were obtained from administrative records of CENAPRECE, specifically, from the Rabies Control Program.

[a] Approximated value based on the proportion of reported attacks at home.

[b] Proportion was estimated from laboratory tests carried out and the number of dogs isolated.

[c] Sistema de Información en Salud (SIS), 2016

[d] About 20% of the dogs entered a rabies-control center (CENAPRECE: 749, including dog food, stationery, salaries of the administrative and medical staff), and 80% of the dogs were quarantined in their homes. Home quarantine required four visits per dog, which takes an average of about four hours. The technician's salary who visits the dog was approximately MX $ 2,500 per month (MX $ 10.49 per hour) if the person graduated from high school, or MX $ 6,700 per month ($ 27.91 per hour) if the person had some college education. This gives a total of MX $ 166.56 and MX $ 446.56 per visit, respectively, assuming that around 70% of visits are made by people with basic education.

Table I. Summary of costs and coverage of dog vaccination campaigns in Mexico, 1990-2015

Year	Vaccinated dogs	Vaccinated dog population (%)	Total costs vaccination campaign	Cost per vaccine
1990	6,190,947	69.42%	87,168,752	14.08
1991	6,917,530	71.92%	92,847,028	13.42
1992	8,336,265	82.97%	108,042,953	12.96
1993	8,616,365	80.35%	110,450,387	12.82
1994	8,860,992	79.57%	130,200,075	14.69
1995	9,773,109	84.69%	145,129,082	14.85
1996	9,659,633	81.59%	130,921,013	13.55
1997	10,257,307	84.05%	133,001,153	12.97
1998	11,144,463	88.65%	137,603,821	12.35
1999	11,826,235	90.87%	142,622,385	12.06
2000	12,238,673	91.49%	145,762,879	11.91
2001	13,105,817	91.92%	154,176,670	11.76
2002	13,871,113	94.14%	171,585,794	12.37
2003	14,320,595	95.21%	180,745,167	12.62
2004	14,355,338	93.95%	184,253,937	12.84
2005	14,848,916	94.33%	189,585,453	12.77
2006	15,102,210	94.51%	197,142,329	13.05
2007	15,317,003	94.55%	206,917,286	13.51
2008	15,658,542	94.60%	218,249,680	13.94
2009	15,828,136	94.17%	247,366,522	15.63
2010	15,840,508	93.05%	250,482,119	15.81
2011	16,508,742	95.57%	264,131,860	16.00
2012	16,430,011	93.43%	268,126,747	16.32
2013	16,527,529	92.39%	297,250,041	17.99
2014	17,076,614	94.49%	318,615,464	18.66
2015	16,649,274	89.86%	323,745,133	19.45

4. Additional results: health indicators of the rabies control program

Table J. Main health indicators of the rabies control program evaluation: human deaths from rabies, the equivalent in years of life potentially lost due to premature death (YLL), and the estimated number of rabid dogs México, 1990-2015

Year	Death with rabies vaccination program			Deaths without rabies vaccination program			No public health intervention‡	
	Humans	YLL	Rabid dogs	Humans	YLL	Rabid dogs	Humans	YLL
1990	60	3,131	7,652	276	14,403	63,026	379	19,785
1991	45	2,367	5,529	448	23,555	64,965	592	31,125
1992	29	1,537	3,588	476	25,210	65,766	625	33,135
1993	20	1,067	2,458	488	26,049	66,246	642	34,226
1994	19	1,019	2,341	497	26,687	66,380	662	35,509
1995	22	1,187	2,748	504	27,176	66,174	676	36,479
1996	15	813	1,869	508	27,525	65,656	668	36,213
1997	20	1,089	2,542	510	27,742	64,881	687	37,383
1998	7	382	906	510	27,850	63,917	687	37,545
1999	3	164	429	508	27,879	62,839	703	38,522
2000	0	0	48	507	27,862	61,726	689	37,917
2001	1	55	146	504	27,829	60,647	644	35,545
2002	0	0	49	503	27,813	59,664	651	36,009
2003	1	55	133	502	27,840	58,827	654	36,278
2004	0	0	5	503	27,934	58,172	664	36,878
2005	2	111	251	505	28,116	57,726	648	36,065
2006	0	0	26	509	28,399	57,499	638	35,600
2007	0	0	0	515	28,792	57,494	676	37,794
2008	0	0	1	523	29,198	57,702	656	36,623
2009	0	0	3	533	29,730	58,105	662	36,906
2010	0	0	1	545	30,399	58,676	680	37,885
2011	0	0	52	559	31,248	59,379	762	42,570
2012	0	0	38	574	32,164	60,172	725	40,623
2013	0	0	48	590	33,194	61,007	711	39,984
2014	0	0	9	606	34,251	61,833	714	40,333
2015	0	0	4	623	35,304	62,598	746	42,305
**Total**	244	12,978	30,874	13,327	734,149	1,601,076	17,241	949,237

Notes. YLL (years of life lost) were estimated based on life expectancy in Mexico, and the rabid dogs' estimate was obtained from CENAPRECE. We did not use time preferences or age weights to be consistent with current practice by the World Health Organization and Global Burden of Disease (GBD)[14] studies. Table J repeats results from Table 2 in the main article with an additional scenario of no public health interventions: no mass rabies vaccination and no PEP. Results combine data from CENAPRECE and estimates from *RabiesEcon* [5], adapted to the dog rabies campaign in Mexico by the CENAPRECE team.

‡ Reflects the number of deaths and YLL if there had been no mass vaccination campaign for dogs or PEP for dog bite victims. The calculation is based on a 0.19 probability of acquiring rabies if the person had been exposed to the virus but does not receive PEP [12].

5. Additional sensitivity analysis

5.1 Alternative scenarios for public health services complementary to dog vaccination

One of the primary sources of uncertainty in this evaluation is estimating the scenario without an annual rabies vaccination campaign for dogs, the counterfactual scenario. It is impossible to know with certainty what would have happened between 1990 and 2015, for example, with the number of investigations by dog bites that CENAPRECE had carried out, the number of dogs under observation or in quarantine, the number of samples analyzed in a laboratory, or the number of people who would have received PEP as a result of the aggression of a dog suspected of rabies. The main article shows our best estimate of the counterfactual scenario based on the available data (reference case).

<u>Main results, reference case (counterfactual scenario)</u>: For our best estimate of what would have happened without mass vaccination, several assumptions were made (also described in the main article). First, considering that there is a higher percentage of dogs with rabies in the scenario without vaccination, we estimated that the number of investigations by dog bite would remain in a similar proportion to 1990, the year in which the massive campaigns of dogs' vaccination began.

To estimate the number of dogs in isolation and quarantine and the number of laboratory investigations in the reference scenario, we added additional rabies exposures estimated with *RabiesEcon*. In the absence of vaccination, we assumed that CENAPRECE would have investigated all attacks by rabid dogs. Experience in rabies prevention and control suggests that the proportion of the population that seeks healthcare when bitten by a suspected rabid dog is more significant the higher the rate of ongoing rabies transmission. With less rabies transmission, the proportion of people receiving PEP also decreases.

<u>Most conservative scenario</u>: We assumed that the annual number of dog bites investigations, quarantine and isolation, laboratory tests, and the percentage of people receiving PEP would have been the same with and without the national vaccination program against rabies in dogs. The assumption in this scenario is that the supply of public health services would be determined by a fixed capacity of the health system in Mexico rather than dog rabies' prevalence.

<u>Less conservative scenario</u>: We assumed that the annual number of dog bites investigations, quarantine and isolation, laboratory tests, and the percentage of people receiving PEP would have progressively increased at constant intervals until reaching 50% more coverage than observed in 1990. Thus, the supply of public health services would progressively increase until it reached 50% more coverage than in 1990 (reference year).

Table K. Sensitivity analysis: most conservative scenario. Main results for the average cost-effectiveness evaluation of the national program of rabies control in Mexico, 1990-2015 (MXN 2015), compared with an estimated counterfactual scenario without mass dog rabies vaccination program, from the government's perspective.

**Indicator**	**No vaccination (counterfactual)**	**Vaccination (current situation)**	**Difference**
**Epidemiologic**			
Total dog rabies cases	1,601,076	30,984	1,570,093
Dog mediated human rabies deaths	13,327	244	13,083
Years of life lost	734,149	12,978	721,171
**Costs (MXN 2015)**			
Dog vaccination campaign	-	4,836,123,729	4,836,123,729
Dog bite investigations	35,295,083	35,295,083	-
Dog isolation and quarantines	497,852,480	497,852,480	-
Laboratory investigations	215,863,562	215,863,562	-
PEP	494,560,020	494,560,020	-
Total	1,243,571,145	6,079,694,874	4,836,123,729
**Average cost-effectiveness 1990-2015**[c]			
Cost per dog rabies case averted	-	3,080	3,080
Cost per human death averted	-	369,656	369,656
Cost per life-year gained	-	6,706	6,706

Notes. The evaluation only considered dog rabies transmission and one annual dog vaccination campaign with no reinforcement. We did not consider sterilization activities. The methods and main assumptions are explained further in the discussion section and the supplementary files.

Table L. Sensitivity analysis: least conservative scenario. Main results for the average cost-effectiveness evaluation of the national program of rabies control in Mexico, 1990-2015 (MXN 2015), compared with an estimated counterfactual scenario without mass dog rabies vaccination program, from the government's perspective.

Indicator	No vaccination (counterfactual)	Vaccination (current situation)	Difference
**Epidemiologic**			
Total dog rabies cases	1,601,076	30,854	1,570,089
Dog mediated human rabies deaths	13,327	244	13,083
Years of life lost	734,149	12,978	721,171
**Costs (MXN 2015)**			
Dog vaccination campaign	-	4,836,123,729	4,836,123,729
Dog bite investigations	53,035,953	35,295,083	-17,740,870
Dog isolation and quarantines	631,250,165	497,852,480	-133,397,685
Laboratory investigations	286,796,145	215,863,562	-70,932,583
PEP	775,463,951	494,560,020	-280,903,931
Total	1,746,546,213	6,079,694,874	4,333,148,661
**Average cost-effectiveness 1990-2015[c]**			
Cost per dog rabies case averted	-	2,760	2,760
Cost per human death averted	-	331,210	331,210
Cost per life-year gained	-	6,008	6,008

Notes. The evaluation only considered dog rabies transmission and one annual dog vaccination campaign with no reinforcement. We did not consider sterilization activities. The methods and main assumptions are explained further in the discussion section and the supplementary files.

Figure A. Sensitivity analysis of cost-effectiveness indicators: incremental cost (with the program - without the vaccination program) for (i) dog rabies cases averted, (ii) per human death averted (in MXN hundreds), (iii) per year of life gained.

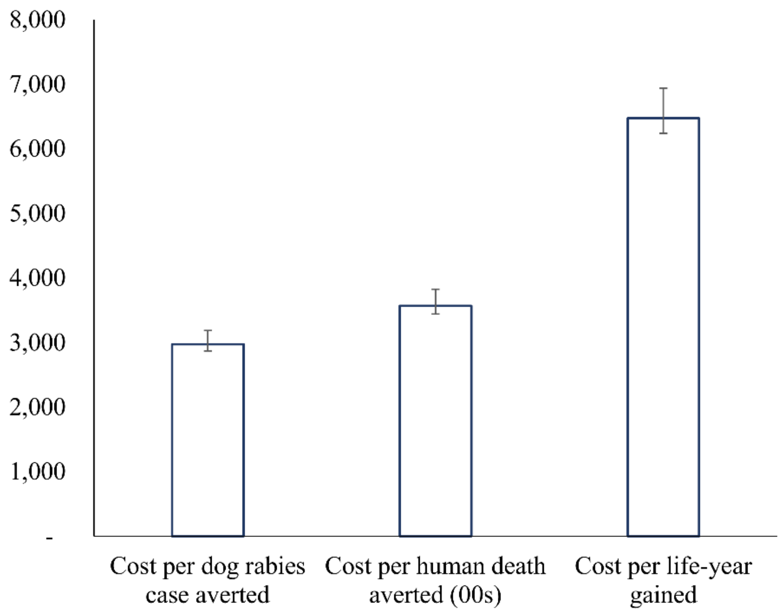

Figure A shows the incremental cost (with vaccination program - without vaccination program) per dog rabies cases averted, per human death averted (in hundreds of pesos), and per year of human life gained. The interval shown by each bar reflects the differences between our best cost estimate, the most conservative scenario, and the least conservative scenario.

The estimates in Tables 3, K, and L, and Figure A suggest that the main results do not change based on the assumptions made about the number of dog bite investigations, quarantine, and isolation of dogs, laboratory tests, and the proportion of people receiving PEP.

5.2 Alternative scenarios for rabies transmission

We defined two additional rabies transmission scenarios to assess how the model could eventually affect our evaluation results: (i) a low rabies transmission and (ii) high rabies transmission. Table M shows the parameters.

Table M. Parameters for the sensitivity analysis in scenarios of low and high rabies transmission, to estimate the counterfactual scenario without annual mass dog vaccination campaigns, Mexico 1990-2015

Dog-dog transmission parameters	Low transmission	High transmission
Number of bites from a dog with rabies to another dog	3.05	5.0
Average number of rabies cases generated by a rabid dog, in a stable state of transmission [a]	1.07	1.75

Notes: [a] Known in epidemiology as basic reproduction number, R_0. Hampson et al. [15] estimate R_0 from time series of rabies incidence in urban and rural contexts, as reported in the scientific literature. In general, the review shows relatively low R_0 but a substantial between-study variation.

6. References in supplementary material

1. Pan American Health Organization. Eliminación de la rabia humana transmitida por perros en América Latina. Análisis de la situación. Washington, DC: World Health Organization, 2005.

2. Vigilato MA, Cosivi O, Knöbl T, Clavijo A, Silva HM. Rabies update for Latin America and the Caribbean. Emerg Infect Dis. 2013;19(4):678.

3. Vigilato MAN, Clavijo A, Knobl T, Silva HMT, Cosivi O, Schneider MC, et al. Progress towards eliminating canine rabies: policies and perspectives from Latin America and the Caribbean. Philosophical Transactions of the Royal Society of London B: Biological Sciences. 2013;368(1623):20120143.

4. World Health Organization. Mexico is free from human rabies transmitted by dogs Geneva: Pan American Health Organization, World Health Organization,; 2020 [cited 2020 November 1]. Available from: https://bit.ly/2J9ZkrY.

5. Borse RH, Atkins CY, Gambhir M, Undurraga EA, Blanton JD, Kahn EB, et al. Cost-effectiveness of dog rabies vaccination programs in East Africa. PLoS Negl Trop Dis. 2018;12(5):e0006490. doi: 10.1371/journal.pntd.0006490.

6. Zinsstag J, Dürr S, Penny M, Mindekem R, Roth F, Gonzalez SM, et al. Transmission dynamics and economics of rabies control in dogs and humans in an African city. Proc Natl Acad Sci. 2009;106(35):14996-5001.

7. Rajeev M, Metcalf CJE, Hampson K. Modeling canine rabies virus transmission dynamics. In: Fooks A, Jackson A, editors. Rabies Scientific Basis of the Disease and Its Management. 4th edition ed: Academic Press; 2020.

8. Jeon S, Cleaton J, Meltzer MI, Kahn EB, Pieracci EG, Blanton JD, et al. Determining the post-elimination level of vaccination needed to prevent re-establishment of dog rabies. PLoS Negl Trop Dis. 2019;13(12):e0007869. doi: 10.1371/journal.pntd.0007869.

9. Instituto Nacional de Estadística y Geografía. Anuario Estadístico y Geográfico por Entidad Federativa 2015 Mexico City: Instituto Nacional de Estadística y Geografía (INEGI),; 2015 [cited 2016 May 18]. Available from: http://internet.contenidos.inegi.org.mx/contenidos/productos/prod_serv/contenidos/espanol/bvinegi/productos/nueva_estruc/AEGPEF_2015/702825077297.pdf.

10. Secretaría de Salud México. Sistema nacional de información en salud Mexico DF: Secretaría de Salud; 2016 [cited 2016 May 18]. Available from: http://www.sinais.salud.gob.mx.

11. Eng T, Fishbein D, Talamante H, Hall D, Chavez G, Dobbins J, et al. Urban epizootic of rabies in Mexico: epidemiology and impact of animal bite injuries. Bull World Health Organ. 1993;71(5):615-24.

12. Shim E, Hampson K, Cleaveland S, Galvani AP. Evaluating the cost-effectiveness of rabies post-exposure prophylaxis: a case study in Tanzania. Vaccine. 2009;27(51):7167-72.

13. Undurraga EA, Meltzer MI, Tran CH, Atkins CY, Etheart MD, Millien MF, et al. Cost-Effectiveness Evaluation of a Novel Integrated Bite Case Management Program for the Control of Human Rabies, Haiti 2014–2015. Am J Trop Med Hyg. 2017;96(6):1307-17. doi: doi:10.4269/ajtmh.16-0785.

14. Murray CJ, Ezzati M, Flaxman AD, Lim S, Lozano R, Michaud C, et al. GBD 2010: design, definitions, and metrics. Lancet. 2012;380(9859):2063-6.

15. Hampson K, Dushoff J, Cleaveland S, Haydon DT, Kaare M, Packer C, et al. Transmission dynamics and prospects for the elimination of canine rabies. PLoS Biology. 2009;7(3):e1000053. doi: 10.1371/journal.pbio.1000053.
